# M7824: A promising new strategy to combat cancer immune evasion

**DOI:** 10.18632/oncoscience.451

**Published:** 2018-08-22

**Authors:** Margaret E. Gatti-Mays, James L. Gulley

**Affiliations:** Laboratory of Tumor Immunology and Biology, Center for Cancer Research, National Cancer Institute, National Institutes of Health, Bethesda, MD 20892, USA

**Keywords:** M7824, PD-L1, transforming growth factor-beta

Checkpoint inhibitors produce durable results across multiple tumors, but only in a subset of patients. There is significant interest in expanding the clinical benefit of checkpoint inhibitors to more patients by developing new types of checkpoint inhibitors including tumor necrosis factor (TNF) receptor superfamilies (TNFRSFs) such as OX40 (also known as CD134; expressed on activated effector T cells and regulatory T cells), 41BB (also known as CD137; expressed on activated T lymphocytes), and glucorticoid-induced TNFR-related protein (GITR; a cell surface receptor constitutive expressed at high levels on regulatory T cells), while regimens combining checkpoint inhibitors with other therapeutic modalities seek to overcome mechanisms of resistance to checkpoint inhibitors.

Higher levels of transforming growth factor-beta (TGF-β) are associated with immune escape, therapy resistance (chemotherapy, radiation, checkpoint inhibitors), and poor outcomes in advanced malignancies [1, 2]. Multiple TGF-β-targeting agents evaluated in the clinic (e.g., fresolimumab, galunisertib, trabedersen) have demonstrated limited efficacy and some toxicity. Inhibiting TGF-β signaling by sequestering TGF-β in the tumor microenvironment (TME) leads to phenotypic changes in nonimmune cells and to enhanced activation of immune cells [3]. Nonresponders to anti-programmed cell death 1/programmed death ligand 1 (PD-1/PD-L1) antibodies have been found to have increased TGF-β signaling in the TME [4, 5]. Thus, it is clear that TGF-β plays an important role in immune resistance to checkpoints and other therapies. However, blockade of only the TGF-β pathway is insufficient to fully engage the immune system and reduce tumor volume.

M7824 (MSB0011359C) is a first-in-class bifunctional fusion protein composed of the extracellular domain of two TGF-β receptor 2 molecules that serve as a TGF-β sequestering or trap molecule fused to a fully humanized monoclonal antibody against PD-L1 (similar to avelumab) [6]. M7824 simultaneously blocks the PD-L1 and TGF-β pathways [3, 5] of immune evasion which are independent yet complementary, with effects on both the adaptive and innate immune systems [7]. Preclinical data demonstrate that M7824 enables immune-cell infiltration and can overcome resistance seen with other PD-L1 antibodies through phenotypic modifications. Early clinical data on M7824 demonstrate an acceptable safety profile and higher responses than expected in heavily pretreated patients with immunologically “cold” tumors [7].

Many mechanisms of resistance to checkpoint inhibitors can be reversed through TGF-β sequestration with M7824. Mariathasan et al. demonstrated that higher levels of TGF-β signaling in stromal cells was associated with immune-excluded tumors [4]. Furthermore, dual inhibition of the TGF-β and PD-L1 pathways leads to a vigorous immune response, with T cell penetration into immune-excluded cancers and tumor regression [4, 8]. Preclinical and clinical studies with M7824 have shown decreased levels of circulating TGF-β [2, 3, 7]. Grenga et al. demonstrated that treatment with M7824 resulted in increased expression of the gene CXCL11, a chemokine involved in trafficking and homing of T cells to tumor [1]. Preclinical studies have shown decreased regulatory T cells, increased early activated and proliferating CD8+ tumor-infiltrating lymphocytes, tumor-infiltrating natural killer (NK) cells, tumor-associated dendritic cells, and mature dendritic cells [3, 5, 6]. Preclinical studies have also demonstrated that M7824 enhanced sensitivity to NK-mediated lysis by antibody-dependent cell-mediated cytotoxicity in tumors that are refractory to chemotherapy, as well as increased sensitivity to TRAIL-mediated lysis [1, 2, 6].

Besides upregulating CXCL11, M7824 also downregulates genes involved with angiogenesis and epithelial-mesenchymal transition [1, 2]. Evaluation of murine models showed a 3-fold increase in the expression of PD-L1, increased major histocompatability complex class II in the TME, and improved antigen presentation when paired with an Ad-TWIST vaccine [2, 3].

A phase 1 trial of M7824 (NCT02517398) has shown promise in advanced, heavily pretreated solid tumors and demonstrated a manageable safety profile comparable to other anti-PD-1/PD-L1 antibodies [7]. While this was a small trial (n = 19), there were signs of clinical efficacy in heavily pretreated patients, with one complete response (human papillomavirus [HPV]+ cervical cancer), two durable confirmed partial responses (pancreatic cancer and HPV+ anal cancer), one near partial response (cervical cancer; HPV status unknown), and two cases of prolonged stable disease in patients with progressing disease at study entry (pancreatic cancer and gastrointestinal carcinoid) [7]. Like other anti-PD-1/PD-L1 antibodies, responses to M7824 appear to be durable, probably due to a distinct immune compartment seen in the TME with a greater CD8+ T-cell or NK-cell response [3, 7].

Early clinical responses and analysis of immune responses after treatment with M7824 suggest improved efficacy compared to traditional anti-PD-1/PD-L1 antibodies. These findings on the immune and nonimmune effects of M7824 make it an attractive candidate for combination with other immuno-oncology agents to better engage, expand, and enable the immune system. Several trials combining therapeutic vaccines across a variety of tumors have either recently opened or are soon to open. An ongoing clinical trial (NCT03315871) is evaluating two therapeutic cancer vaccines (PROSTVAC and CV301) in combination with M7824. In addition, an adaptive-design clinical trial evaluating M7824 in combination with up to three other immunotherapy agents in prostate cancer recently opened at the National Cancer Institute (NCT03493945). Many other trials of M7824 combined with other agents are being planned.

## Abbreviations

GITR: glucorticoid-induced TNFR-related protein
HPV: human papillomavirus
NK: natural killer
PD-1: programmed cell death protein 1
PD-L1: programmed-death ligand 1
TGF-β: transforming growth factor-beta
TME: tumor microenvironment
TNF: tumor necrosis factor
TNFRSFs: tumor necrosis factor receptor superfamilies
